# An optimized workflow for improved gene expression profiling for formalin-fixed, paraffin-embedded tumor samples

**DOI:** 10.1186/2043-9113-3-10

**Published:** 2013-05-03

**Authors:** Marlene Thomas, Manuela Poignée-Heger, Martin Weisser, Stephanie Wessner, Anton Belousov

**Affiliations:** 1 Pharma Research and Early Development (pRED), Roche Diagnostics GmbH, TR-H, Bldg 231/206a, Nonnenwald 2, 82377 Penzberg, Germany

**Keywords:** Biomarker, Breast cancer, Gene, HER2, Microarray

## Abstract

**Background:**

Whole genome microarray gene expression profiling is the ‘gold standard’ for the discovery of prognostic and predictive genetic markers for human cancers. However, suitable research material is lacking as most diagnostic samples are preserved as formalin-fixed, paraffin-embedded tissue (FFPET). We tested a new workflow and data analysis method optimized for use with FFPET samples.

**Methods:**

Sixteen breast tumor samples were split into matched pairs and preserved as FFPET or fresh-frozen (FF). Total RNA was extracted and tested for yield and purity. RNA from FFPET samples was amplified using three different commercially available kits in parallel, and hybridized to Affymetrix GeneChip® Human Genome U133 Plus 2.0 Arrays. The array probe set was optimized *in silico* to exclude misdesigned and misannotated probes.

**Results:**

FFPET samples processed using the WT-Ovation™ FFPE System V2 (NuGEN) provided 80% specificity and 97% sensitivity compared with FF samples (assuming values of 100%). In addition, *in silico* probe set redesign improved sequence detection sensitivity and, thus, may rescue potentially significant small-magnitude gene expression changes that could otherwise be diluted by the overall probe set background.

**Conclusion:**

In conclusion, our FFPET-optimized workflow enables the detection of more genes than previous, nonoptimized approaches, opening new possibilities for the discovery, validation, and clinical application of mRNA biomarkers in human diseases.

## Background

Cancer is a broad label, covering a variety of tumor subtypes that are associated with different prognoses and different responses to the various treatment options available. Traditionally, solid tumors are studied by histopathologic assessment. However, as cancer is a heterogeneous molecular disease, classification of tumor types should ideally be accompanied by molecular biology techniques.

Gene expression profiling is a technology for identifying the genes that are active in a given sample of cells or tissue. This technique enables profiling of genes which are differentially expressed in tumors, thereby providing diagnostic and prognostic information for molecular subclassification of tumors [1-4]. Gene expression profiling of tumor samples using microarray technology has promised more accurate prognoses and has the potential to improve therapeutic options for patients [5,6]. Results from such studies have already been translated into clinical practice. For instance, gene expression profiling has identified a powerful predictor of the outcome of disease in young patients with breast cancer; this outperformed standard systems based on clinical and histologic criteria [7]. However, more research is needed before gene expression profiling can become routine in the clinic.

The application of retrospective microarray analyses to long-term archived tissues – as well as to fresh biopsy samples that reflect the present disease state – could provide assistance in a number of areas. It may help in the elucidation of disease mechanisms, in detection of novel therapeutic targets, and in identification of prognostic markers. Analysis of samples collected during randomized trials can also be used for identifying predictive markers [8]. Quantitative determination of genome-wide expression levels via microarray technology also provides a powerful approach for comparative analysis of normal and neoplastic tissues.

Whole genome microarray gene expression profiling is the ‘gold standard’ for the discovery of prognostic and predictive genetic markers for human cancers [9]. Most gene expression profiling studies use fresh-frozen (FF) tissue, which yields high-quality RNA [9]. However, the use of FF samples is currently not directly applicable to tumor types that are characterized using histologic analysis, as the worldwide standard in this setting is formalin fixation followed by embedding the fixed tissue in paraffin. Collection and storage of fresh material would, therefore, require a change in standard clinical protocols as well as substantial additional logistical efforts, greater demands on resources, and increased compliance for all those involved with the collection and processing of clinical trial samples. Formalin fixation and paraffin embedding is the clinical ‘gold standard’ for tissue preservation as it preserves tissue architecture [10] and allows the long-term storage of tissue in archival banks. Millions of such archived samples provide a resource that can be correlated with long-term clinical outcomes [11]. As most diagnostic biopsies and surgically excised samples available in tissue banks are formalin-fixed, paraffin-embedded tissue (FFPET) samples, there is a lack of suitable research material available for gene expression profiling. Using archived specimens increases the probability of identifying clinically relevant biomarkers, so subjecting archived FFPET samples to gene expression profiling could assist in the elucidation of disease mechanisms, detection of novel therapeutic targets, and identification of prognostic markers. Furthermore, FFPET samples offer the most efficient means of determining tumor profile (by detecting correlation between gene expression profiles and covariates such as immune cell infiltration, and necrotic or fibrotic cell content). It is, therefore, important that all techniques used for examining tissue samples are optimized for FFPET samples.

It is well documented that formalin fixation and paraffin embedding degrades RNA in tissue samples [10,12,13]. RNA derived from FFPET samples is not only partially hydrolyzed but also chemically modified: formalin reacts with nucleotides, leading to the introduction of methylol groups into the bases. These groups tend to further react and form intra- and inter-molecular methylene bridges in RNA, DNA, and protein [13]. These alterations can interfere with enzyme activity during the process of reverse transcription, which is a critical step in microarray sample processing. However, several researchers have demonstrated that it is possible to extract RNA from FFPET samples [14], although the poor quality of RNA derived from FFPET samples means that reliable quantification of gene expression is subjected to serious limitations. In addition, differences in the formalin fixation techniques, such as ischemia time and the fixation protocol used, such as drying times, as well as storage time and conditions, can all lead to differences in results for gene expression [10,12,13].

There are also differences between the commercially available kits used for RNA extraction and processing, and so the use of different kits may lead to differences in the results obtained. The results of RNA profiling are expected to be platform-specific. Proper annotation of probes and tailored data analysis also help in controlling bias and reducing the variability of the results.

Archived specimens have no corresponding FF samples to act as comparator. Therefore, it is important to ascertain the validity of any results obtained from using FFPET samples. Research comparing gene expression profiling in FF and FFPET samples has already been done using various extraction and amplification techniques and a variety of tissues, and it has been concluded that valid data can be obtained from FFPET samples [15-22], although results have been variable. Gene expression profiling protocols for use with FFPET samples continue to be explored, but further research is needed to optimize and standardize RNA extraction and amplification techniques and data analysis methods, so that robust conclusions can be drawn from the results.

The aim of this study is to determine whether FFPET samples are suitable for significant profiling analysis using a new, optimized workflow and data analysis method. The Affymetrix GeneChip® Human Genome U133 Plus 2.0 Array platform was used and the results from three different commercially available RNA amplification kits were compared. The three RNA amplification kits were assessed in parallel and the expression signatures of matched FFPET material and native FF tissue from the same tumor were compared. If it can be shown that gene expression profiling is possible from FFPET samples, and that the profiling can detect stable genes that display good signals, then using this optimized system could reveal many more genes than previously reported from FFPET samples; this would be an important step in disease biomarker discovery.

## Methods

### Tissue samples

Sixteen breast tumor samples (>12 months old) were purchased from Asterand, Inc. (see asterand.com) for information concerning ethical conduct, eight were estrogen receptor-positive (ER+) and eight were estrogen receptor-negative (ER–). The ER status was confirmed by immunohistochemistry and all samples consisted of more than 70% of tumor cells.

### Tissue preparation

Each tumor sample was divided into mirror sections, half being FF and half being formalin-fixed and paraffin-embedded using standard protocols. Total RNA was extracted from 10 consecutive sections cut in each direction from the mirrored area. FFPET sections measured 10 μm and FF sections measured 15 μm. The purified RNA from each fixation type was pooled. The first and the last section for both FF and FFPET samples were hematoxylin and eosin stained to exclude any significant variations in the investigated tumor area.

### Total RNA extraction

RNA extractions were performed according to the manufacturers’ instructions as follows:

For FF cryosections, the RNeasy® Mini Kit and DNA digestion step (QIAGEN AG) were used*.* For FFPET sections, the High Pure FFPET RNA Isolation Kit® from Roche Diagnostics was used.

### RNA quality evaluation

The yield and purity of RNA was determined using a NanoDrop 1000 Spectrophotometer (Thermo Scientific). RNA integrity was measured using a Bioanalyzer (Agilent Technologies) with either the RNA Nano or Pico Kit, depending on sample concentration. The RNA Integrity Number (RIN) and average length of the RNA were used for sample characterization.

mRNA integrity in FFPET samples was measured using a semiquantitative reverse transcription-polymerase chain reaction assay (rtPCR). This assay is based on quantitative amplification of 5′- and 3′-end sequences of the housekeeping gene β-actin. Calculating the ratio of 3′ to 5′ amplicons allows for assessment of mRNA integrity: intact RNA transcripts exhibit a ratio of 1, while degraded RNA increases the ratio. A Transcriptor First Strand cDNA Synthesis Kit (Roche) was used to prepare cDNA from 100 ng of total RNA, using an anchored oligo(dT)-primer followed by a subsequent amplification step with the SYBR Green I Master on a LightCycler® 480 instrument (Roche Applied Science). Genomic DNA residues were determined via rtPCR using a LightCycler® 480, with 100 ng purified RNA as template material.

### Amplification and labeling

RNA samples from FF tissue were amplified using the 3′ IVT Express Kit (Affymetrix). Fluorescence labeling and fragmentation were carried out according to the manufacturer’s protocol.

FFPET-derived RNA was amplified with either the GeneChip® Expression 3′-Amplification Two-Cycle Kit (Affymetrix), the WT-Ovation™ FFPE System V2 (NuGEN), or the TransPlex® Complete Whole Transcriptome Amplification Kit (Sigma/Rubicon Genomics).

Biotin labeling of the cDNA generated using the Affymetrix technique was performed using the 3’ IVT Express Kit (Affymetrix), while cDNA generated with the NuGEN technique was performed using the FL-Ovation cDNA Biotin Module V2 (NuGEN). Biotin labeling of cDNA generated with the Sigma/Rubicon Genomics technique was performed using the ULS™ aRNA Labeling Kit with Biotin-ULS for Affymetrix Genechips (Kreatech Diagnostics).

### Hybridization and processing

Hybridization of labeled probes on the Affymetrix GeneChip Human Genome U133 Plus 2.0 Array were conducted using the GeneChip® Hybridization, Wash, and Stain Kit (Affymetrix).

Target preparation, hybridization of samples to microarrays, and washing of microarrays were conducted according to the respective manufacturers’ manuals. For this workflow a GeneChip Fluidics Station 450 and a GeneChip Scanner 3000 7G were used.

### Data analysis

All analyses were performed using C and R statistical data analysis developed at Roche. Extensive quality control of the wet-lab performance, such as background signal and signal gradients, masking of spots (outliers), and estimation of optical noise, was followed by gene expression index estimation. This was performed for every amplification method separately with a modified version of the Robust Multichip Average (RMA) renormalized matched log algorithm [23].

**Figure 1:**
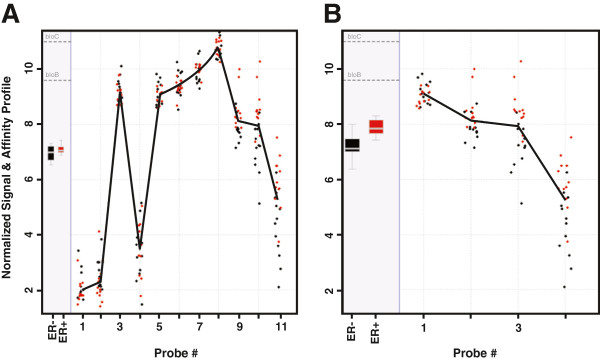
Plot showing (A) typical poor-quality Affymetrix probe set and (B) the revised probe set used in this study.* OY axis corresponds to probe-dependent signal component, also known as affinity profile. Signals are normalized by RMA method. Black dots = ER–, red dots = ER+. * The set of 11 probes shown in Figure 1A contains seven probes that either do not match the genome and transcriptome (probes 1 to 4) or that are not sufficiently specific for their target sequence and may, therefore, detect multiple genes (probes 6 to 8). Concentrating on an unambiguous part of the probe set (Figure 1B) significantly improves the power of the statistical tests used (eg, a *t*-statistic of 3.07 comparing expression in estrogen receptor-negative versus expression in estrogen receptor-positive tumors as opposed to a *t*-statistic of 0.98 when the original Affymetrix probe set is used).

To minimize annotation errors, the original Affymetrix probe set design of the array was not taken into account. Instead, every probe on the array (604,258 probes in total) was first mapped against the latest human genome build and transcriptome databases: UniGene, RefSeq, and EMBL [24,25]. The probes that passed this first quality control step were considered for the revised probe set design. In order to make the results of the present analysis comparable across multiple similar studies published so far, an *in silico* array design was created that was as close to the initial Affymetrix design as possible, but which excluded misdesigned or misannotated probes. Consequently, the original probe set was divided into three subsets: a perfectly matching and unique part (unique: 43,098 probe sets in total); a subset consisting of probes that appear to match transcripts of multiple genes (nonunique: 3,981 probe sets); and the remaining probe sets that failed to match against either DNA or any known transcript (mismatched: 11,660 probe sets). An example of a poor-quality original probe set is shown in Figure 1. The value of the proposed *in silico* revision is supported by the results of a *t* test to determine the likelihood of an association between ER gene expression patterns and ER status: whereas no significant association was detected using the original Affymetrix probe set design (*P* = 0.35), a strong association was detected using the optimized probe set (*P* = 0.008). On close examination, the original ER probe set can be seen to contain four nonmatching probes and three nonunique probes.

### Ingenuity pathways analysis

Genes controlling the ER signaling pathway were identified in FF and FFPET samples using Ingenuity Pathways Analysis (Ingenuity Systems).

## Results

### RNA yield and integrity

All samples yielded sufficient RNA to proceed to microarray processing, and most RNA samples were sufficiently concentrated to enable an accurate assessment of integrity using the RIN tool (lower limit = 25 ng/μl). RIN values were obtained for all but five samples (all FFPET-derived). As expected, RIN values indicated more degradation of RNA in FFPET samples (RIN range, 1.8–2.8) than in FF samples (RIN range, 5.4–9.0). This observation was supported by 3′/5′ ratios: ratios ranged from 4.2 to 151.3 in FFPET samples and from 1.0 to 1.5 in FF samples [Table 1].

**Table 1:** RNA concentration, RNA integrity number and 3′/5′ ratios for fresh-frozen and formalin-fixed, paraffin-embedded tissue samples

**Sample**	**FF samples**			**FFPET samples**		
	**RNA conc. (ng/μL)**	RIN*	**3′/5′ ratio**	**RNA conc. (ng/μL)**	**RIN**	**3′/5′ ratio**
1	153	7.8	1.0	258	2.1	4.2
2	27	9.0	1.1	71	2.2	6.8
3	40	6.2	1.4	156	2.5	24.2
4	98	7.0	1.2	87	1.8	7.4
5	49	8.6	1.0	127	NA	5.1
6	73	7.3	1.0	96	NA	90.5
7	326	8.6	1.0	467	NA	90.8
8	87	6.8	1.2	168	6.2	44.3
9	34	5.4	1.5	74	NA	151.3
10	55	8.3	1.0	100	NA	90.8
11	23	6.9	1.2	53	2.6	9.0
12	120	7.9	1.0	162	2.8	72.2
13	58	7.7	1.1	71	2.6	17.1
14	110	6.9	1.1	178	2.5	11.3
15	121	7.8	1.1	219	2.3	16.6
16	175	7.1	1.2	349	2.3	16.1

*FF*, fresh-frozen; *FFPET*, formalin-fixed, paraffin-embedded tissue; *NA*, not assessable; *RIN*, RNA integrity number.

* RNA integrity is gauged using the RIN, which indicates the intactness of the sample by assigning a grade from 1 to 10, 10 being the most intact.

**Figure 2:**
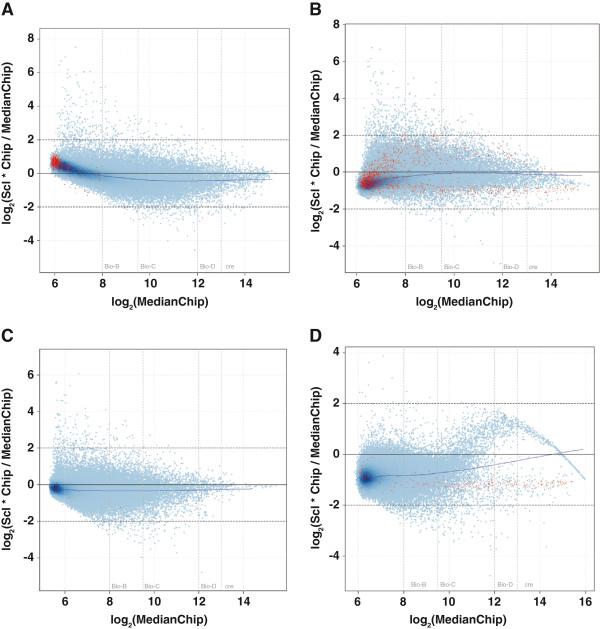
**Pre-normalization microarray plots.** (**A**) Fresh-frozen tissue, (**B**) Formalin-fixed, paraffin-embedded tissue (FFPET) processed using the Affymetrix Two-Cycle kit, (**C**) FFPET tissue processed using the NuGEN kit, and (**D**) FFPET tissue processed using the Sigma/Rubicon Genomics kit.

The microarray plots also demonstrate that total RNA from the FFPET samples showed more evidence of degradation compared with total RNA from the FF samples [Figure 2]. Of the three amplification kits compared, the NuGEN amplification kit demonstrated the closest correlation to the ‘gold standard’ FF results, while the Sigma/Rubicon Genomics amplification kit produced most deviated data [Table 2].

**Table 2:** Quality measures of microarray gene expression values*

	**Correlation of gene expression between matched FF and FFPET samples**	**Percentage present call**^**†**^	**Percentage probe sets with expression > LOB**	**Percentage genes with non-consistent expression across multiple probe sets**
	**Median (25%, 75% quantiles)**	**Median (25%, 75% quantiles)**		
FF:	N/A	49.7 (47.3, 50.6)	51.4	17.4
FFPET: Affymetrix	0.78 (0.72, 0.81)	33.4 (21.8, 38.1)	36.2	19.4
FFPET: NuGEN	0.88 (0.84, 0.89)	47.2 (44.1, 58.1)	52.7	17.9
FFPET: Sigma/Rubicon	0.48 (0.47, 0.52)	22.9 (22.1, 24.6)	16.4	28.0

*FF*, fresh-frozen; *FFPET*, formalin-fixed, paraffin-embedded tissue; *LOB*, limit of blank; *N/A*, not applicable.

* For the genes represented by at least two probe sets the analysis of consistency of probe set expression is performed. As an overall measure of the consistency we have estimated the percentage of genes for which the Pearson correlation coefficient between expression of all pairs of related probe sets did not exceed 0.5.

^†^ As defined by the Affymetrix algorithm.

### Percentage present calls

An alternative and more direct way of estimating the percentage of genes expressed in a sample is by counting the number of probe sets whose expression values exceed the limit of blank (LOB). LOB marks the lowest level of expression that can definitely be attributed to sample-derived human mRNAs. We estimated the LOB value to be the 95% percentile of the expression of the ‘complete mismatch’ probe sets. The ‘complete mismatch’ probe sets were composed of the probes that failed to match both genomic sequence and known mRNA and EST transcripts, even when up to three mismatches were tolerated. Table 2 shows that quantification of probe sets achieved using the NuGEN RNA amplification kit is similar to that achieved with ‘gold standard’ FF amplification. For genes represented by at least two probe sets an analysis is performed to determine the consistency of sequence detection between probe sets. As an overall measure of the consistency we estimated the percentage of genes for which the Pearson correlation coefficient between related probe sets did not exceed 0.5. Once again, gene expression data obtained from FFPET samples processed using the NuGEN kit were most closely comparable with FF samples.

### Sensitivity and specificity – whole genome

**Figure 3:**
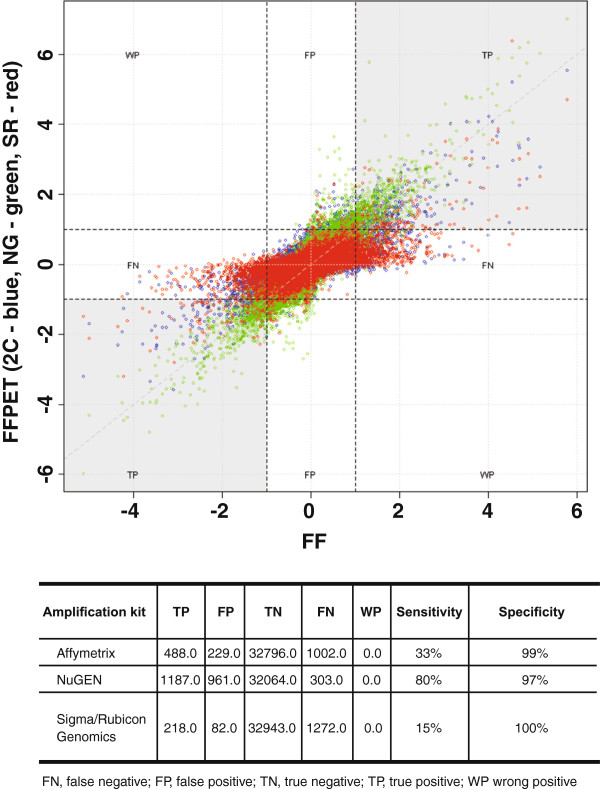
**Estrogen receptor status of FFPET samples: sequence detection sensitivity and specificity by different methods.** Each point of a particular color in the plot represents the effect size (difference between mean expression in ER+ and ER– samples) found in FF samples and FFPET samples using a particular workflow. Different quadrants of the plot are marked so as to assist in classifying the findings. For example, false negative (FN) findings are those which are present in FF but absent in FFPET. Presence and absence is decided based on the observed effect size with the widely accepted threshold of 2-fold change (up or down). One particular case, referred to as a wrong positive finding (WP), not observed in our experiments, is when a relevant (>1 in absolute value) effect in FF stays relevant but changes its direction in FFPET.

The FF sample results, as the ‘gold standard’, were assumed to have 100% sensitivity and specificity for gene detection. The sensitivity of a particular FFPET profiling workflow was defined as the percentage of the probe sets found ‘relevant’ by both the FF and the FFPET method used. The threshold for relevance in the FF samples was taken as a twofold increase or decrease in gene expression of ER+ and ER–. Once again, the best overall results were obtained using the NuGEN kit. Using the NuGEN method, sensitivity, at 80%, was increased by 47 percentage points over the Affymetrix Two-Cycle kit, and 65 percentage points over the Sigma/Rubicon Genomics kit. Specificity, defined as the percentage of the probe sets that showed relevant differential expression in FFPET samples but not in FF material, was slightly lower using the NuGEN kit, at 97%, compared with 99% for the Affymetrix Two-Cycle kit and 100% for Sigma/Rubicon Genomics [Figure 3]. Similar results were obtained with a subset of genes from the ER signaling pathway (data not shown).

Independent analysis of the expression of the gene *DDX5* was undertaken as an internal control for validating the results of the microarray data. *DDX5* has been proposed to be a very stable housekeeping gene, showing greater correlation between quantitative rtPCR and microarray data than *GAPDH*[26]. This analysis showed that processing FFPET samples using the NuGEN amplification kit provided the closest comparison with the FF sample processing. The results obtained using the Affymetrix Two-Cycle kit showed a wide scatter, unlike the FF sample, and results obtained with the Sigma/Rubicon Genomics kit failed to show expression of *DDX5* (data not shown).

Signal detection sensitivity and specificity generally depends on effect size. For this experiment, the sensitivity and specificity were estimated independently for the couple of subsets of genes that have effect size in a predefined range. The results show that the larger the expected effect is in FF samples, the easier it is to see the same effect in FFPET samples. Dynamic sensitivity and specificity estimations show the correlation of fold changes, such as a greater than twofold increase between ER+ and ER–, in the FF samples with the sensitivity of the corresponding FFPET sample.

### Principal component analysis

Principal component analysis is a useful dimensionality reduction technique that has been used to visualize patterns in multivariate gene expression data [27]. Samples are represented by several principal components rather than by the individual gene expression values obtained from all probe sets. These components, sometimes called ‘super genes’, are linear combinations of probe set expression values and are constructed in such a way as to account for as much of the variability in the data as possible.

**Figure 4:**
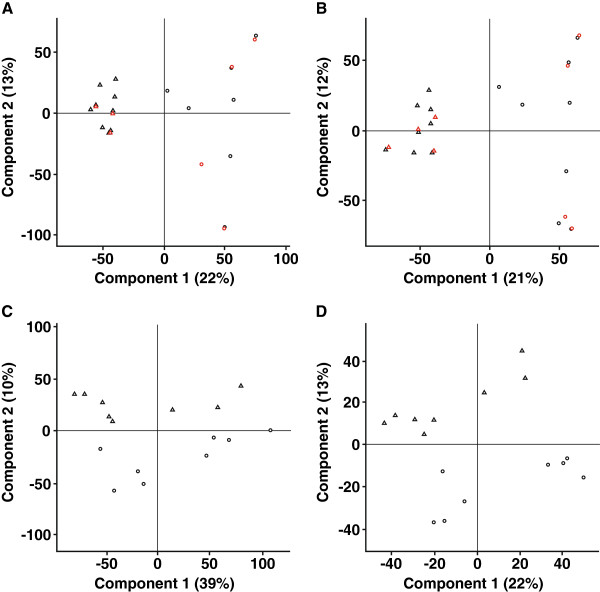
**Principal components analysis plots.** (**A**) Fresh-frozen tissue, (**B**) Formalin-fixed, paraffin-embedded tissue (FFPET) samples processed using the NuGEN RNA amplification kit, (**C**) FFPET samples processed using the Affymetrix Two-Cycle RNA amplification kit, and (**D**) FFPET samples processed using the Sigma/Rubicon Genomics RNA amplification kit. Note the extremely similar shape of fresh-frozen and FFPET data clusters associated with the NuGEN workflow. The NuGEN kit also provides the most parsimonious description: in order to capture the effect of estrogen receptor (ER) status on gene expression, both the Two-Cycle and the Sigma/Rubicon Genomics kit would require at least two leading principal components. Circles and triangles represent ER– and ER+ samples correspondingly. Technical replicates are shown in red.

Comparison of the principal component analysis plots [Figure 4] for matched FF and FFPET samples with ER+ and ER– status reinforces the similarity between data obtained from FFPET samples processed using the NuGEN amplification kit and the FF samples. FF and FFPET samples displayed similar clustering of data points, as well as similar sample order, overall shape, and data distribution pattern.

### Ingenuity pathway analysis

The canonical ER pathway was extracted from the Ingenuity Knowledge Base and the fold changes in ER pathway genes were mapped against those pathways [see Additional file 1, Additional file 2, Additional file 3, Additional file 4]. The microarray expression data obtained for the ER pathway matched known ER pathway biology, indicating biologic plausibility for microarray data. While both nonoptimized and optimized probe sets detected evidence of a strong biologic effect associated with ER gene expression, a nonoptimized probe set would not have detected many of the potentially significant but smaller-magnitude changes in gene expression occurring within the tissue. This effect is evident when sequence-detection sensitivity values are compared across the ER-related probe sets [Figure 3]. This result demonstrates that it is possible to elucidate the same ER signaling network from both FF and FFPET samples.

## Discussion

Gene expression profiling classifies individual tumors by their gene expression patterns and may also describe and predict therapeutic resistance and sensitivity patterns. Profiling in several cancers, such as breast cancer, colon cancer, lymphoma, leukemia, and melanoma [3], has already identified molecular subclasses of tumors. Identification of tumor subtypes may be predictive for prognosis or response to drug therapy [6,7,28-31].

The potential of routine gene expression profiling to predict clinical outcomes for cancer patients has yet to be determined. The Evaluation of Genomic Applications in Practice and Prevention Working Group stated in 2009 that there was “insufficient evidence to make a recommendation for or against the use of tumor gene expression profiles to improve outcomes in defined populations of women with breast cancer” [32]. Clearly, more work needs to be done to translate promising research findings into clinically relevant results.

Comparison of FFPET sample-derived gene expression profiles with associated clinical outcome data could provide an invaluable resource for the investigation of mechanisms of response and resistance to cancer therapeutics. This will, however, require a suitably reliable method for extracting RNA from these samples and an FFPET-optimized workflow for use in gene expression profiling studies. Two recent studies performed using archived FFPET core biopsies from the NOAH trial (neoadjuvant trastuzumab with chemotherapy in patients with locally advanced/inflammatory breast cancer) [33] have demonstrated that biomarkers predictive of treatment response can be identified from FFPET processed with an optimized workflow such as that described here [34,35]. These findings speak to a vast untapped potential for biomarker discovery due to the abundance of archived tissues available for gene expression profiling.

FFPET samples could also be used prospectively to identify biomarkers in clinical trials. Biomarkers discovered via microarray gene expression profiling could then be applied clinically using more widely available technologies, such as immunohistochemistry or quantitative rtPCR. Recently, researchers conducting a multicenter randomized phase III trial in colon cancer have suggested that translational studies based on FFPET samples should be included in prospective clinical trials [8].

Methods and protocols for RNA isolation from formalin-fixed tissues have been available for over 20 years [13] but results have been variable. Research continues to develop optimized protocols for formalin fixation [36] and RNA extraction methods [37].

The work reported here shows that there is no loss of data if RNA amplification and *in silico* probe design protocols are optimized towards FFPET samples (which were >12 months old). Specificity of gene detection was 99% using the Affymetrix method, 97% using the NuGEN method, and 100% using the Sigma/Rubicon Genomics method. The gene detection sensitivity results obtained from FFPET samples varied depending on which of the three different amplification kits was used. The combination of the NuGEN RNA purification/amplification method with the Affymetrix Human Genome U133 Plus 2.0 Array resulted in 80% sensitivity with 97% specificity. This agrees with Linton et al. [21] who also reported 80% sensitivity, assuming FF sensitivity to be 100%, using the same NuGEN amplification and labeling system when comparing sarcoma FFPET with FF samples. Williams et al. [22] have also demonstrated the superiority of the NuGEN method over the Affymetrix method. It should be noted that newly developed FFPET amplification systems (such as Affymetrix Sensation Plus®) may increase the sensitivity and specificity of signal detection from FFPET. Furthermore, detailed feasibility studies with FFPET samples that are >2 years old should be carried out to provide further evidence that archival tissue is suitable for gene expression profiling.

It is also important to show that the genes identified in this new workflow are an accurate reflection of the biology of the sample. The Ingenuity Pathways Analysis shows that the same ER signaling pathway is identified regardless of whether the sample was FF or FFPET, and that therefore the FFPET data obtained are a true reflection of the biology of the tissue sample.

The correlation between gene expression profiling results obtained from FF and FFPET samples is greatly improved using the new array design and data processing methods reported here. A comprehensive gene expression profile is necessary to both the discovery of novel biomarkers and the development of new disease subclassifications. Our optimized sample processing workflow and data analysis method enables the detection of genes that cannot be detected using a nonoptimized approach. The gene detection rate reported here for FFPET-derived samples is similar to that obtained using FF samples, demonstrating the importance of optimal RNA amplification and raw data processing with refined probe set alignments, which could facilitate the discovery of biomarkers from FFPET samples. In addition, other commercial microarrays are now available which may overcome the current limitations associated with the Affymetrix GeneChip.

The ‘percentage of genes present’ call is a useful tool to evaluate the overall quality of gene expression data. However, if the percentage present call is to be used as a quality control measure, then it may be advisable to set a precise threshold for discarding poor-quality samples so that potentially useful data – such as single gene profiles obtained from individual samples – are not unnecessarily lost. Consequently, the homogenous distribution of percentage present calls in the sample population may be a more useful quality control measure than percentage present call alone. Using this method, a sample with a markedly low percentage present call could be identified as an outlier even in a genome-wide analysis.

The different techniques and kits used in gene expression profiling render direct comparisons with previously reported data problematic. Although the redesigned probe set used here is as close to the original Affymetrix design as possible, comparisons with previously reported data sets using the Affymetrix array may also be problematic for several reasons. For example, differences in FFPET techniques between laboratories result in variable degradation of sample RNA. In addition, the age of the sample can impact results, although Tanney and Kennedy have reported that it is possible to extract good-quality transcriptional data from samples as old as 17 years using the Roche High Pure FFPE extraction kit and NuGEN WT-Ovation FFPE RNA amplification system [14]. The quality of gene expression data can also vary according to the RNA extraction and amplification methods used.

Handling and fixation protocols vary between centers and evolve over time, so the genetic variation identified between tumors could be an artifact of tissue processing rather than a reflection of tumor biology. To overcome this, it may be important to pool samples from several centers to ensure that they represent the population under consideration as a whole [14]. This would require careful study design to avoid adding confounding factors.

It is also important to ensure that the sample size selected is sufficient for the study to be adequately powered to detect differences in gene expression. Most tumor studies are aimed at finding genes with at least twofold higher or lower expression than found in normal tissues. Power analysis for this study (data not shown) demonstrated that a protocol with 10 samples per category was 40% powered to detect a twofold change in expression, but could reliably detect a threefold change.

## Conclusion

In conclusion, optimizing processing methods for use with FFPET samples, together with optimized array design and data processing, should provide a clinically useful data set from the large archive of FFPET samples already available, and from FFPET samples routinely collected in clinical trials. In addition, *in silico* probe set redesign may enable the detection of potentially significant but smaller-magnitude changes in gene expression that may be diluted by the standard probe set background. The methods presented here have the potential to enable a vast expansion in the quantity of gene expression data available for analysis alongside other data, such as immunohistochemistry results. These findings suggest that it is crucial to apply the most optimal sample processing approach concerning RNA amplification and subsequent raw data processing, using the refined probe set alignment. Finally, this workflow could be beneficial for identifying potentially novel disease markers, which have been missed in previous data sets due to sensitivity or specificity issues. It is important to further invest in optimizing the gene expression profiling work flow to make sure that RNA information is interpreted correctly for the discovery, validation, and clinical application of mRNA biomarkers in human diseases.

## Competing interests

All authors are employed by Roche Diagnostics GmbH, Germany.

## Authors’ contributions

MT and MW contributed to the scientific background, study design, planning of experiments and manuscript writing. AB contributed statistical support, data analysis, Affymetrix chip re-design and manuscript writing. SW carried out sample preparation, RNA extraction, sample analysis and QC and contributed to manuscript writing. MPH contributed to study design, planning of experiments, sample analysis and QC and manuscript writing. All authors read and approved the final manuscript.

## Supplementary Material

Additional file 1 Estrogen receptor signaling comparison of FF and FFPET NuGEN results: FF samples. Click here for file

Additional file 2 Estrogen receptor signaling comparison of FF and FFPET NuGEN results: FFPET samples processed using NuGEN workflow. Click here for file

Additional file 3 Estrogen receptor signaling comparison of FF and FFPET NuGEN results: FFPET samples processed using Sigma/Rubicon workflow. Click here for file

Additional file 4 Estrogen receptor signaling comparison of FF and FFPET NuGEN results: FFPET samples processed using Affymetrix Two-Cycle workflow. Click here for file
