# Supplementary figures and images for: An optimized workflow for improved gene expression profiling for formalin-fixed, paraffin-embedded tumor samples
Source: J Clin Bioinforma. 2013 May 3;3:10. doi: 10.1186/2043-9113-3-10 (PMC3660273; doi:10.1186/2043-9113-3-10)

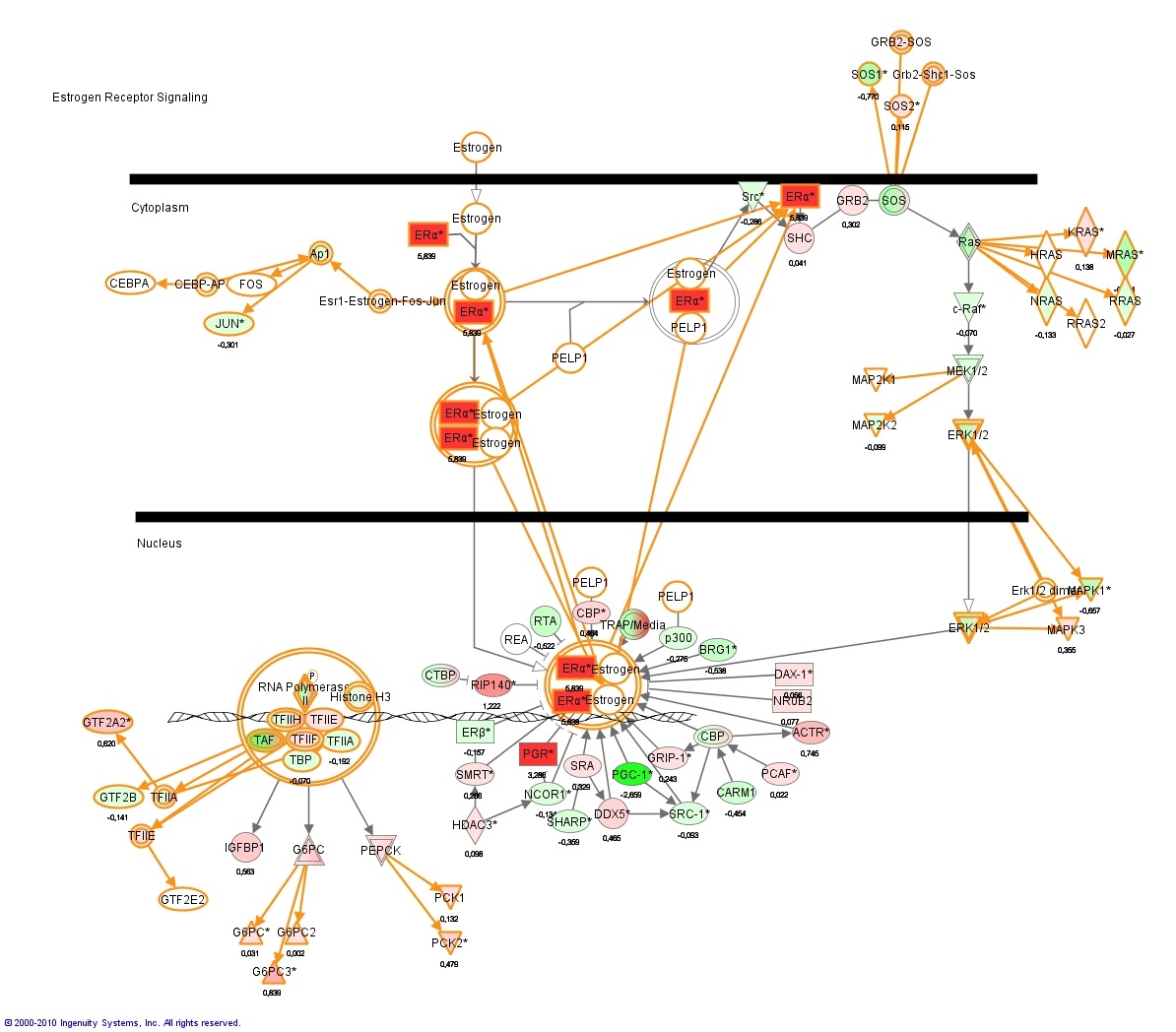

Supplement: Additional file 1 — Estrogen receptor signaling comparison of FF and FFPET NuGEN results: FF samples. [file 2043-9113-3-10-S1.jpeg]

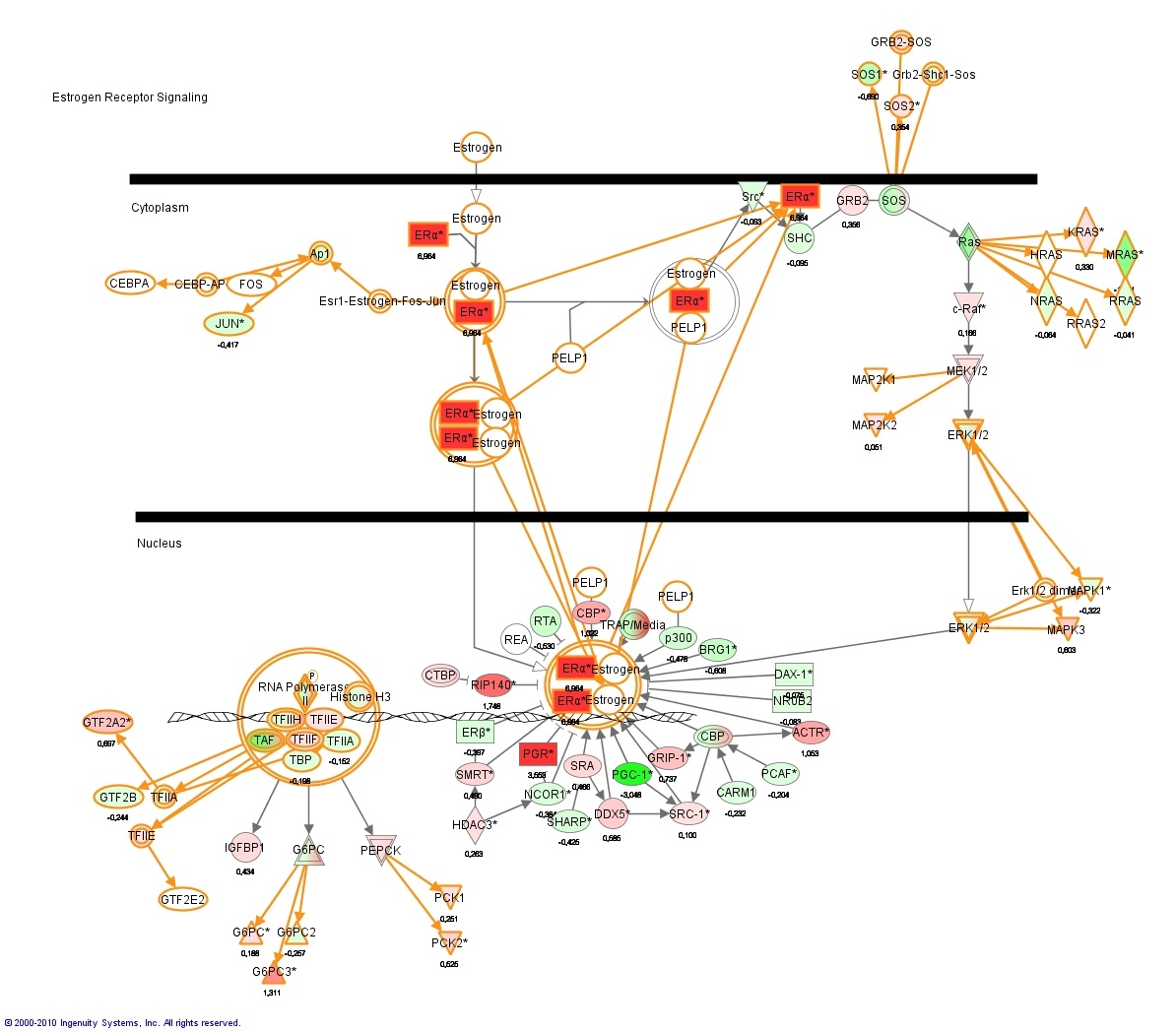

Supplement: Additional file 2 — Estrogen receptor signaling comparison of FF and FFPET NuGEN results: FFPET samples processed using NuGEN workflow. [file 2043-9113-3-10-S2.jpeg]

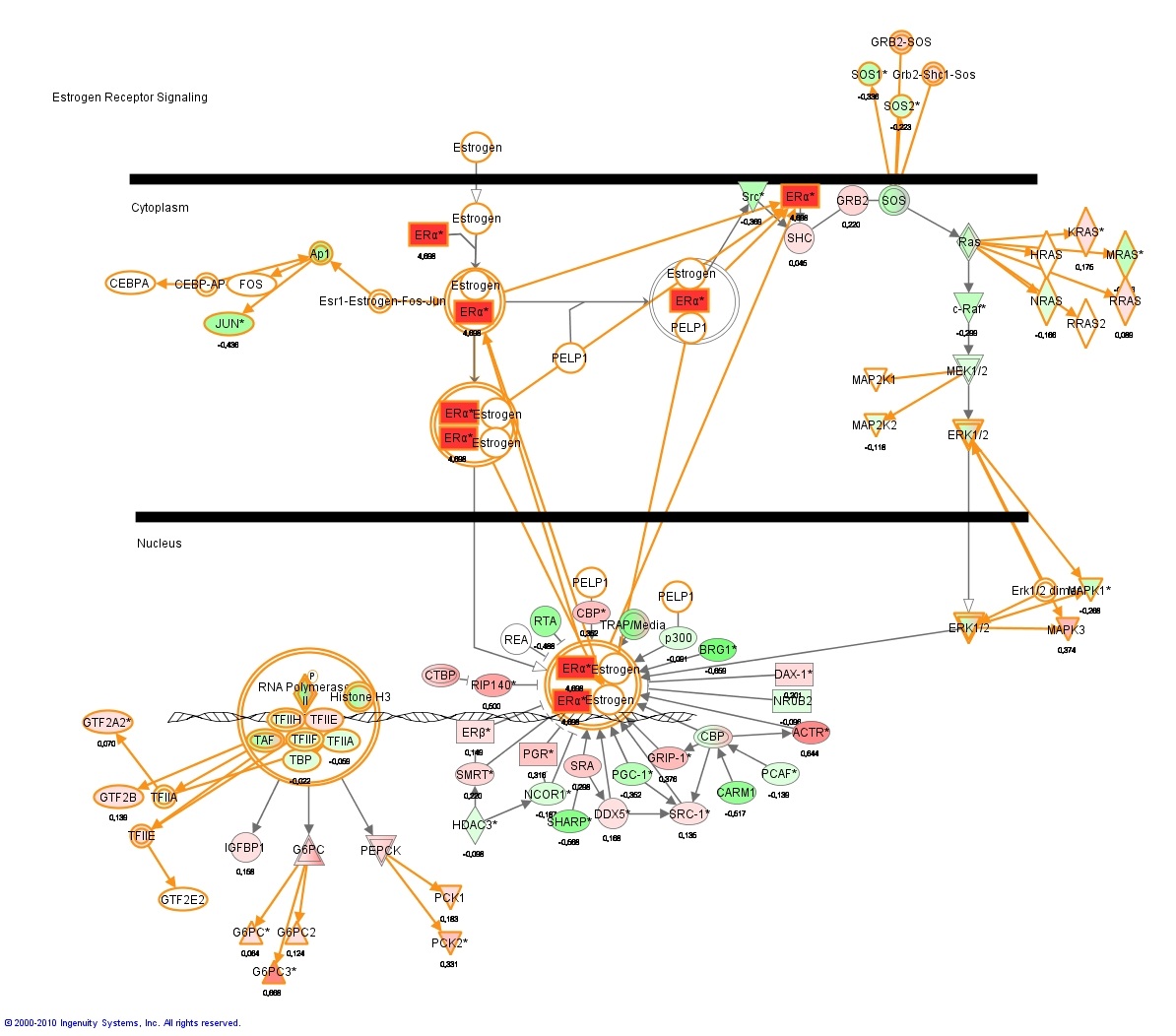

Supplement: Additional file 3 — Estrogen receptor signaling comparison of FF and FFPET NuGEN results: FFPET samples processed using Sigma/Rubicon workflow. [file 2043-9113-3-10-S3.jpeg]

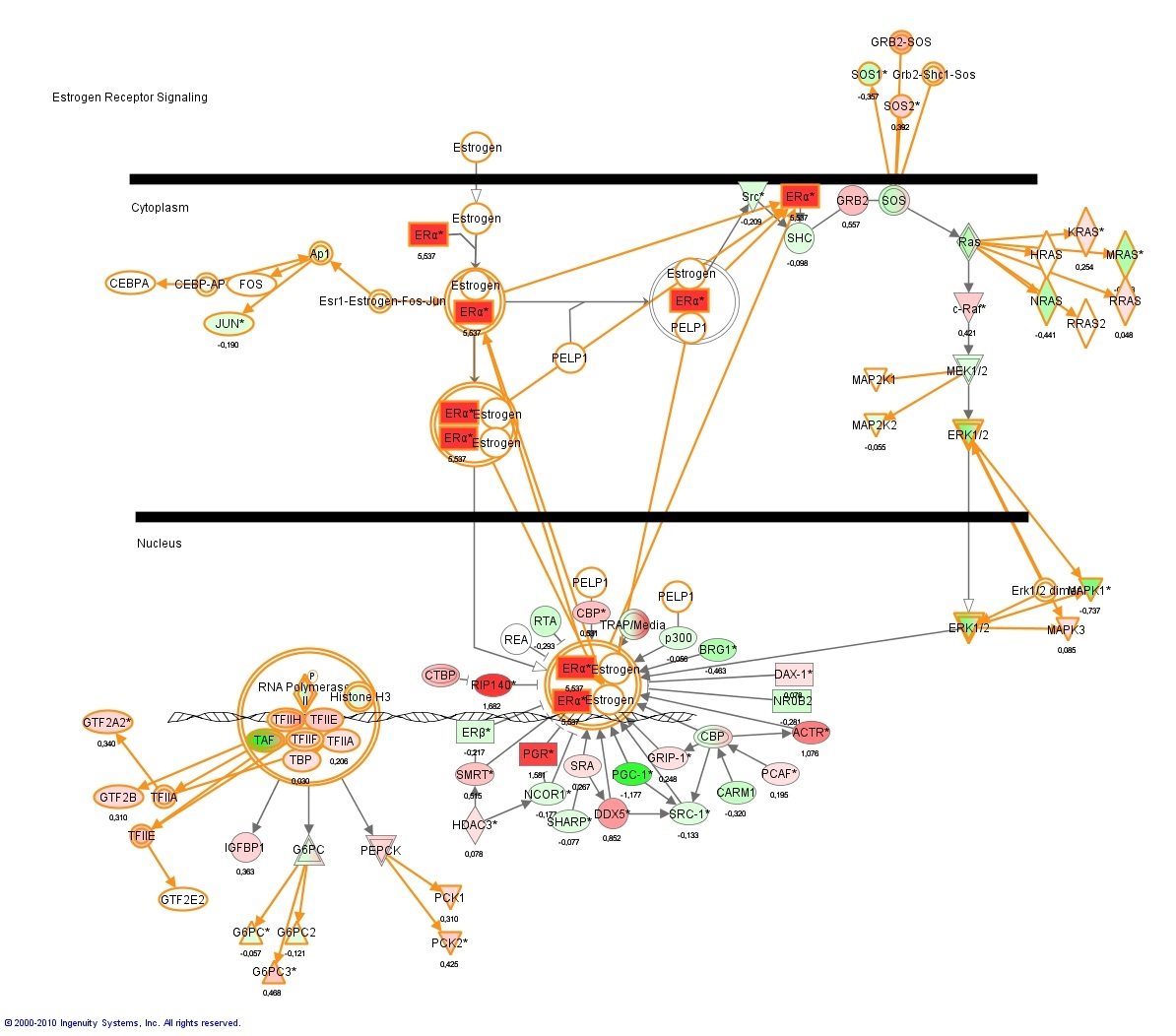

Supplement: Additional file 4 — Estrogen receptor signaling comparison of FF and FFPET NuGEN results: FFPET samples processed using Affymetrix Two-Cycle workflow. [file 2043-9113-3-10-S4.jpeg]
